# Breast cancer-associated metastasis is significantly increased in a model of autoimmune arthritis

**DOI:** 10.1186/bcr2345

**Published:** 2009-07-30

**Authors:** Lopamudra Das Roy, Latha B Pathangey, Teresa L Tinder, Jorge L Schettini, Helen E Gruber, Pinku Mukherjee

**Affiliations:** 1 Department of Immunology, Mayo Clinic School of Medicine, 13400 E. Shea Blvd., Scottsdale, Arizona-85259, USA; 2 Department of Biology, University of North Carolina at Charlotte, 9201 University City Blvd., Charlotte, NC-28223, USA; 3 Department of Orthopedic Surgery, Carolinas Medical Center, 1543 Garden Terrace, Charlotte, NC-28232, USA

## Abstract

**Introduction:**

Sites of chronic inflammation are often associated with the establishment and growth of various malignancies including breast cancer. A common inflammatory condition in humans is autoimmune arthritis (AA) that causes inflammation and deformity of the joints. Other systemic effects associated with arthritis include increased cellular infiltration and inflammation of the lungs. Several studies have reported statistically significant risk ratios between AA and breast cancer. Despite this knowledge, available for a decade, it has never been questioned if the site of chronic inflammation linked to AA creates a milieu that attracts tumor cells to home and grow in the inflamed bones and lungs which are frequent sites of breast cancer metastasis.

**Methods:**

To determine if chronic inflammation induced by autoimmune arthritis contributes to increased breast cancer-associated metastasis, we generated mammary gland tumors in SKG mice that were genetically prone to develop AA. Two breast cancer cell lines, one highly metastatic (4T1) and the other non-metastatic (TUBO) were used to generate the tumors in the mammary fat pad. Lung and bone metastasis and the associated inflammatory milieu were evaluated in the arthritic versus the non-arthritic mice.

**Results:**

We report a three-fold increase in lung metastasis and a significant increase in the incidence of bone metastasis in the pro-arthritic and arthritic mice compared to non-arthritic control mice. We also report that the metastatic breast cancer cells augment the severity of arthritis resulting in a vicious cycle that increases both bone destruction and metastasis. Enhanced neutrophilic and granulocytic infiltration in lungs and bone of the pro-arthritic and arthritic mice and subsequent increase in circulating levels of proinflammatory cytokines, such as macrophage colony stimulating factor (M-CSF), interleukin-17 (IL-17), interleukin-6 (IL-6), vascular endothelial growth factor (VEGF), and tumor necrosis factor-alpha (TNF-alpha) may contribute to the increased metastasis. Treatment with anti-IL17 + celecoxib, an anti-inflammatory drug completely abrogated the development of metastasis and significantly reduced the primary tumor burden.

**Conclusions:**

The data clearly has important clinical implications for patients diagnosed with metastatic breast cancer, especially with regards to the prognosis and treatment options.

## Introduction

Metastasis is regulated not only by intrinsic genetic changes in malignant cells, but also by the microenvironment. Several studies have demonstrated that sites of chronic inflammation are often associated with the establishment and growth of various malignancies [1]. A common inflammatory condition in humans is autoimmune arthritis (AA) that causes inflammation and deformity of the joints. Other systemic effects associated with AA include increased cellular infiltration and inflammation of the lungs and blood vessels (vasculitis), and weakening of the bones (osteoporosis). Although AA and cancer are different diseases, some of the underlying processes that contribute to the disorders of the joints and connective tissue that characterize AA also affect cancer progression and metastasis. In addition, the immune system appears to play an overseer's role in both diseases as reviewed by Ziegler [2]. The most striking link between the two diseases came from a long-term community-based prospective study of the influence of inflammatory polyarthritis (IP) in cancer incidence and survival [3]. The authors reported that inflammatory arthritis increases the risk of dying from cancer (at least double the risk of the general population). Several studies have also reported statistically significant risk ratios between AA and various malignancies including breast, lung, hematopoietic, non-melanotic skin, kidney, and colon [4-6].

Despite this knowledge, which has been available for a decade, there has been minimal research linking arthritis with metastatic breast cancer. It has never been questioned if a site of chronic inflammation linked to AA creates a milieu that attracts tumor cells to home and grow in the inflamed site. The lungs and bones are frequent sites of breast cancer metastasis [7]. The preference of breast cancer cells to grow in the bone and lung is underscored by the fact that 65 to 75% of patients with advanced disease develop bone or lung metastasis [8]. Yet, it is not known why and how breast cancer cells prefer to colonize these organs. There are no methods to predict the risk of breast cancer-associated metastasis and current treatments have notable limitations. We hypothesize that chronic inflammatory milieu and osteoclastic bone resorption caused by AA and the lung inflammation associated with it may influence the recruitment, retention, and proliferation of tumor cells in the bone and lungs.

In this study, we determined if chronic inflammation in the bones and lungs induced by AA contribute to increased breast cancer-associated bone and lung metastasis. We have used a recently established animal model of spontaneous autoimmune arthritis known as SKG mice. These mice are on the Balb/c background and carry a mutation of the gene encoding a SH2 domain of ZAP-70, a key signal transduction molecule in T cells, and spontaneously develop T cell-mediated chronic AA [9]. The mutation impairs positive and negative selection of T cells in the thymus, leading to thymic production of arthritogenic autoimmune CD4^+ ^T cells. The mice succumb to symmetrical joint swelling beginning in the small joints of the digits and progressing to larger joints, accompanied by severe synovitis with formation of pannus invading and eroding adjacent cartilage and subchondral bone.

Genetic deficiency of IL-6, IL-1, or TNF-α inhibit development of AA in SKG mice [10], similar to the effects of anticytokine therapy in human arthritis [11]. These clinical and immunopathological characteristics of AA in these mice make the strain a suitable model for testing our hypothesis. When these arthritic mice were induced to develop metastatic mammary gland tumors, a significant increase in lung and bone metastasis were observed compared with the non-arthritic mice. Furthermore, the severity of arthritis was amplified by factors associated with the metastatic compared with the non-metastatic tumor cells. We have identified some of the key pro-inflammatory factors that may partially contribute to the increased incidence of secondary metastasis. Overall, our data suggests a novel link between AA-induced inflammation and secondary metastasis associated with breast cancer.

## Materials and methods

### Mice

SKG mice have been established from a closed breeding colony of Balb/c mice [9]. Two sets of SKG breeding pairs were purchased from CLEA International (Tokyo, Japan) and were maintained in our animal facility. All protocols were approved by the Mayo Clinic Internal Animal Care Review Committee.

### Induction of arthritis and tumor inoculation

Two month old mice were given a single intraperitoneal (ip) injection of 2 mg zymosan A in 100 μl of 0.15 M sodium chloride (NaCl) per mouse [12] and joint swelling was macroscopically examined starting at 14-days post zymosan A treatment. Thirty days post zymosan A treatment, more than 95% of the mice develop polyarthritis in small and large joints. At this time, mice were injected with 1 × 10^6 ^(in 100 μl of PBS) syngeneic breast cancer cells (4T1: metastatic or TUBO: non-metastatic) in the mammary fat pad. Age-matched SKG mice without zymosan A were used as the pro-arthritic model, and Balb/c mice were used as the non-arthritic controls. As per Institutional Animal Care and Use Committee regulations, we sacrificed the mice when tumors in the mammary fat pad reached 10% of the body weight. All mice were injected with the same number of tumor cells. When tumors in one group of mice reached more than 10% of their body weight, all mice were sacrificed and analysis was conducted. In our study the timepoint at which the mice were analyzed for metastasis was four weeks after tumor inoculation.

Zymosan A was purchased from Sigma-Aldrich, USA (St Louis, MO, USA). A 1% solution of zymosan was made in 0.15 M NaCl, placed in a boiling water bath for one hour, centrifuged for 30 minutes at 4000 rpm and the residue suspended evenly in the 0.15 M NaCl to the desired concentration. It is established that the glucose polymer B-1,3-D-glucans (B-glucans), the main constituents of zymosan A, are responsible for the arthritogenic effect [12].

### Scoring of joint swelling

Joint swelling was monitored by inspection and scored as follows: 0 = no joint swelling; 0.1 = swelling of one finger joint; 0.5 = mild swelling of wrist or ankle; 1.0 = severe swelling of wrist or ankle. Scores for all fingers of forepaws and hindpaws, wrists and ankles were totalled for each mouse. This method is followed according to previously published protocol [9].

### Cell culture

The 4T1 mammary carcinoma cell line was purchased from The American Type Cell Culture Collection (Manassas, VA, USA) and the TUBO mammary carcinoma cell line was generously provided by Dr Joseph Lustgurten, Mayo Clinic College of Medicine. 4T1 and TUBO cells were maintained in RPMI-1640 medium supplemented with 10% FBS, 1% Glutamax-1 and 1% penicillin-streptomycin. Cells were maintained at log phase at 37°C with 5% carbon dioxide. 4T1 is a highly metastatic breast cancer cell line derived from a spontaneously arising BALB/c mammary tumor. TUBO is a cloned cell line established from a mammary carcinoma of the Her2-neu transgenic mice also on the Balb/c background. TUBO is considered to be a nonmetastatic cell line.

### Measurement of circulating cytokines

The RayBio^® ^Custom Mouse Cytokines Antibody Array kit was purchased from RayBiotech (Norcross, GA, USA) and used according to the manufacturer's instructions. Briefly, after blocking with 1 × blocking buffer (provided by the manufacturer), membranes were incubated for 1.5 hours with the experimental serum (10-fold diluted with 1 × blocking buffer). The membranes were washed and incubated with biotin-conjugated antibodies for 1.5 hours. The membranes were washed again and incubated with streptavidin-conjugated horseradish peroxidase for two hours, washed, and developed using an enhanced chemiluminescent substrate for horseradish peroxidase. Chemiluminescence was detected using a EpiChemi3^® ^Darkroom imaging system and LabWorks^® ^densitometry software (both from UVP Bioimaging, Upland, CA, USA). Data was corrected for background signal and normalized to positive controls using RayBio^® ^Analysis Tool software (UVP Bioimaging, Upland, CA, USA).

### Histology

Lungs were formalin fixed in 10% neutral-buffered formalin (pH 6.8 to 7.2) for a minimum of 24 hours. Paraffin-embedded blocks were prepared by the Histology Core at The Mayo Clinic and 4-micron thick sections were cut for H&E staining and for immuno-staining. To determine macrophage infiltration in the lungs, F4/80 (Abcam, Cambridge, MA, USA; CI:A3-1) antibody was used at 1:50 dilution and incubated overnight at 4°C followed by DAKO goat anti-rat secondary (1:100 dilution; Dako North America, Carpinteria, CA, USA). For neutrophil staining, the standard protocol provided by the Naphthol AS-D Chloroacetate Esterase, (Sigma, St Louis, MO, USA; Cat: # 91C-1Kit) was used. We recognize that CAE also may stain for other myeloid cells such as mast cells and macrophages; however, neutrophils stain purple and have the typical morphology as shown in the magnified image. The kit used to stain neutrophils is most commonly used to detect neutrophils in tissue sections that have been paraffin embedded (as indicated in the kit fact sheet). Slides examined under light microscopy and pictures taken at 200× magnification.

For bones, the fore limb and hind limb were dissected from the mice and immersed in 10% neutral-buffered formalin (pH 6.8 to 7.2) overnight. For decalcification, Cal-Rite (Richard Allan Scientific, Kalamazoo, MI, USA), a formic acid decalcification agent was used for about 72 hours followed by the conventional processing method. Masson trichome staining was used to determine levels of osteoclasts. Cyclo-oxygenase-2 (COX-2) and pancytokeratin (Santa Cruz Biotechnologies, Santa Cruz, CA, USA) antibodies were used at 1:50 and incubated overnight at 4°C followed by the DAKO anti-goat secondary (Dako North America; 1:100 dilution) for 45 minutes at room temperature for COX-2. For pancytokeratin staining, DAKO anti-mouse secondary (Dako North America) was used at 1:100 for 45 minutes at room temperature. 3, 3"–diaminobenzidine was used as the chromogen and hematoxylin was used as counterstain. Slides were examined under light microscopy and pictures taken at 200× magnification.

#### Faxitron imaging

The Faxitron is a 160-kVp x-ray machine that was adapted from an x-ray imaging unit through modifications to facilitate experimental irradiation and imaging. The Faxitron model CP160 (Faxitron X-Ray Corp., Wheeling, IL, USA) is a commercially available x-ray tube machine that is designed for animal irradiation [13]. The analysis was conducted by a Mayo Clinic radiologist and lately in Carolinas Medical Center within the Department of Orthopedic Surgery.

#### Invasion assay with bronchoalveolar lavage fluid

Mice were sacrificed by carbon dioxide inhalation, a tracheal cannula was inserted, and bronchoalveolar lavage (BAL) fluid was collected by lavage of the lungs, three times with 1 ml of cold PBS. BAL cells were pelleted by centrifugation and the supernatant collected for tumor invasion assays.

Invasion of 4T1 was tested using a standard trans-well matrigel assay [14]. The inserts (8.0 μM Falcon 353097) were coated with 5 μl of 7.6 μg/μl growth factor-reduced marigel (BD Biosciences, San Jose, CA, USA) diluted 1:5 in serum free RPMI (SF) media. Thirty minutes post incubation at 37°C, 500 μl BAL in SF media was added into a 24-well plate. Complete RPMI media with 10% FCS was used as a positive control and SF media served as the negative control. In to 300 μl SF media, 5 × 10^4 ^4T1 cells were added on the matrigel coated insert. Fifteen hours post incubation at 37°C, non-invaded cells were harvested from the top of the insert. Inserts were inverted and stained with 30 μl of crystal violet (0.5% crystal violet/20% methanol) for 5 to 10 minutes, rinsed with d-H_2_0 and left to dry. The membranes were cut and added to 200 μl 10% acetic acid solution in a 96-well plate, incubated for 10 minutes, removed and read on a spectrophotometer at 562 μM. The percentage (%) of cells invaded = (average reading of the sample/average reading of the control) × 100.

#### Study design for the IL-17 treatment

To test the efficacy of anti-IL-17 antibody treatment on breast cancer-associated metastasis, three month old SKG mice were injected with 1 × 10^6 ^(in 100 μl of PBS) 4T1 cells in the mammary fat pad. When the tumors were 0.2 g or more, three ip injections of 5 μg/ml of anti-IL17 antibody (BD Pharmingen, San Diego, CA, USA; Cat#560268) once a week was administered. Celecoxib (20 mg/kg in 100 μl 10% DMSO) was gavaged starting at the same time as the IL-17 antibody but was given daily until sacrifice. One week after the last injection, mice were sacrificed. Untreated SKG mice challenged with 4T1 cells and the SKG challenged with 4T1 cells and injected with 5 μg/ml rat immunoglobulin (Ig) G1 control antibody (BD Pharmingen, Cat# 554682) in 100 μl PBS were used as controls. Experimental groups were: 1) SKG + 4T1 (celecoxib only); 2) SKG + 4T1(IL-17 only); 3) SKG + 4T1(IL-17+celecoxib); 4) SKG + 4T1 (control antibody + DMSO); and 5) SKG + 4T1 (no treatment).

### Statistical analysis

Student's t-test was used for comparing the level of significance between the experimental groups. Correlation coefficient was determined using the JMP statistical discovery software (SAS Institute Inc., Cary, NC, USA).

## Results

### Metastatic breast cancer cells may contribute to the severity of arthritis in the SKG mice

**Figure 1:**
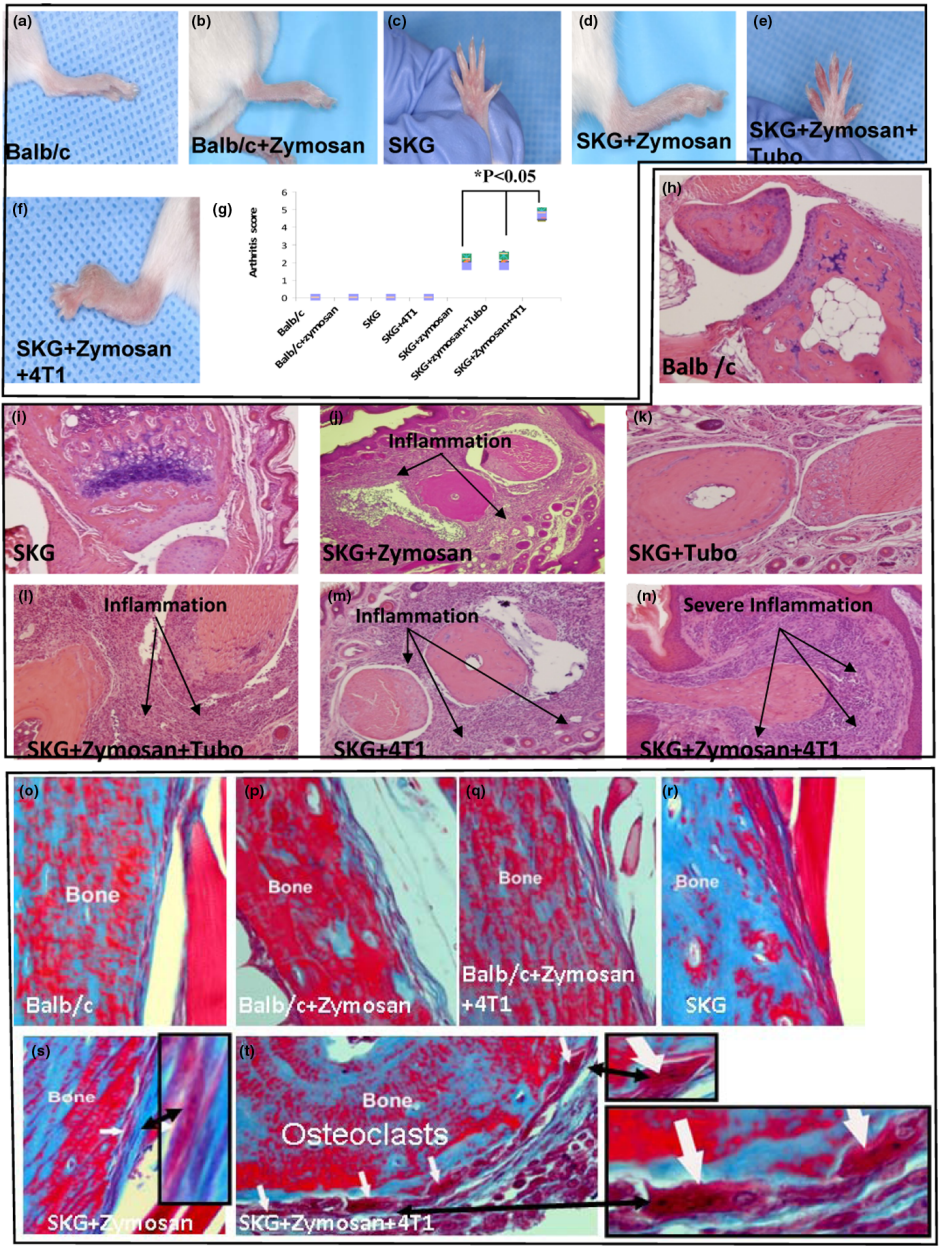
Induction of arthritis in SKG mice. (**a to f**) Images of the hind and fore limbs **(a) **control Balb/c mice (no inflammation); **(b) **Balb/c mice+Zymosan (no inflammation); **(c) **SKG mice (no inflammation); **(d) **SKG mice + Zymosan A (moderate inflammation); **(e) **SKG mice +Zymosan A + TUBO (moderate inflammation); **(f) **SKG mice + Zymosan A + 4T1 cells (severe inflammation). **(g) **Scoring of joint swelling: Compared with SKG mice + Zymosan A or SKG mice +Zymosan A + TUBO, a significant increase in joint swelling was observed in SKG mice injected with Zymosan A and challenged with 4T1 cells (*P *< 0.05). **(h to n) **H&E staining of sections from the joints: **(h) **Balb/c mice; **(i) **SKG mice; **(j) **SKG mice + Zymosan A without tumor challenge (moderate inflammation); **(k) **SKG mice + TUBO cells; **(l) **SKG mice + Zymosan A + TUBO cells showing some erosion of articular cartilage; **(m) **SKG mice + 4T1 cells showing severe inflammation in the phalangeal joints; **(n) **SKG mice + Zymosan A + 4T1 cells showing severe synovial hyperplasia and erosion of articular cartilage and bone in phalangeal joints with severe inflammation; **(o to t) **Masson trichome staining of sections from the bones: **(o) **Balb/c mice; **(p) **Balb/c mice + Zymosan A; **(q) **Balb/c mice +Zymosan A + 4T1; **(r) **SKG mice; **(s) **SKG mice + Zymosan A (arrow represents osteoclast); **(t) **SKG + Zymosan A + 4T1 (multiple osteoclasts marked with arrows). The H&E images of the joints were taken at 200 × magnification and the Masson Trichome bone images taken at 400 × magnification.

In pathogen-free facilities, the SKG mice remain pro-arthritic with no macroscopic signs of joint swelling until treated with zymosan A (a yeast cell wall extract) [12]. Within 30 to 45 days of zymosan treatment, there is clearly macroscopic evidence of joint swelling in the fingers and in the fore and hind limbs (Figures 1a to 1f). SKG, SKG + zymosan (30-days post zymosan), and Balb/c control mice were challenged with 1 × 10^6 ^syngeneic breast cancer cells (4T1 being metastatic and TUBO being non-metastatic) in the mammary fat pad. Compared with the Balb/c ± zymosan and the pro-arthritic SKG mice (Figures 1a to 1c), the joint swelling was two-fold higher in the SKG + zymosan mice ± TUBO tumors (Figures 1d and 1e) and five-fold higher in the SKG + zymosan mice bearing the 4T1 tumors (Figure 1f). Importantly, the SKG + zymosan mice bearing the 4T1 tumors had a two-fold increase in joint swelling compared with the same mice bearing the TUBO tumors or no tumors (Figure 1f compared with Figure 1d and 1e), suggesting that the metastatic 4T1 breast cancer cells may have the potential to augment the severity of arthritis. This is further elucidated in Figure 1g, where the arthritis score in SKG + zymosan + 4T1 mice was significantly higher than in the SKG + zymosan + TUBO mice (*P *< 0.01); while the arthritis score in the SKG + zymosan + TUBO mice remained exactly the same as SKG + zymosan mice without any tumor (Figure 1g) suggesting that TUBO cells did not affect the severity of arthritis in these mice. There were no macroscopic signs of arthritis detected in the SKG + 4T1 mice (Figure 1g). Similar results were seen in eight mice per experimental group. Of note is that none of the Balb/c controls with or without zymosan or SKG without zymosan showed any signs of inflammation (Figures 1a to 1c and 1g).

Histological examination of the bone sections clearly showed that compared with the non-tumor-bearing SKG and the TUBO tumor-bearing SKG mice, the 4T1 tumor-bearing SKG mice had severe inflammation with a high degree of cellular infiltration in the joints (Figures 1i, k, and 1m). Similarly, higher severity of synovial hyperplasia and erosion of articular cartilage in the phalangeal joints of the hind limb along with severe cellular infiltration was found in the 4T1 tumor-bearing SKG + zymosan mice (Figure 1n) compared with the joints of control SKG + zymosan mice with no tumors (Figure 1j) or in the TUBO-bearing SKG + zymosan mice (Figure 1l). It must be acknowledged that although all SKG + zymosan mice showed high infiltration, the severity of infiltration was highest in the SKG + zymosan + 4T1 mice (Figures 1j, l, and 1n). Inflammation was absent in control Balb/c mice, while in the SKG and SKG + TUBO mice low-level infiltration was evident (Figures 1h, i, and 1k). High cellular infiltration in the 4T1-bearing SKG + zymosan mice was associated with increased bone destruction as evidenced by the increased osteoclasts in these mice (Figure 1t) as compared with SKG + zymosan (Figure 1s) or SKG without tumors (Figure 1r). There were no detectable osteoclasts in any of the Balb/c mice (Figures 1o to 1q) or TUBO injected mice (data not shown). SKG + 4T1 mice showed very similar osteoclast expression as SKG + zymosan + 4T1 (data not shown). Taken together these data suggest that the metastatic 4T1 breast cancer cells may contribute to the vicious cycle of osteolytic destruction. Several sections from eight mice per experimental group were examined with similar results. Sections of the distal hind limb joints are shown.

### Significant increase in lung metastasis in the arthritic versus non-arthritic mice

**Figure 2:**
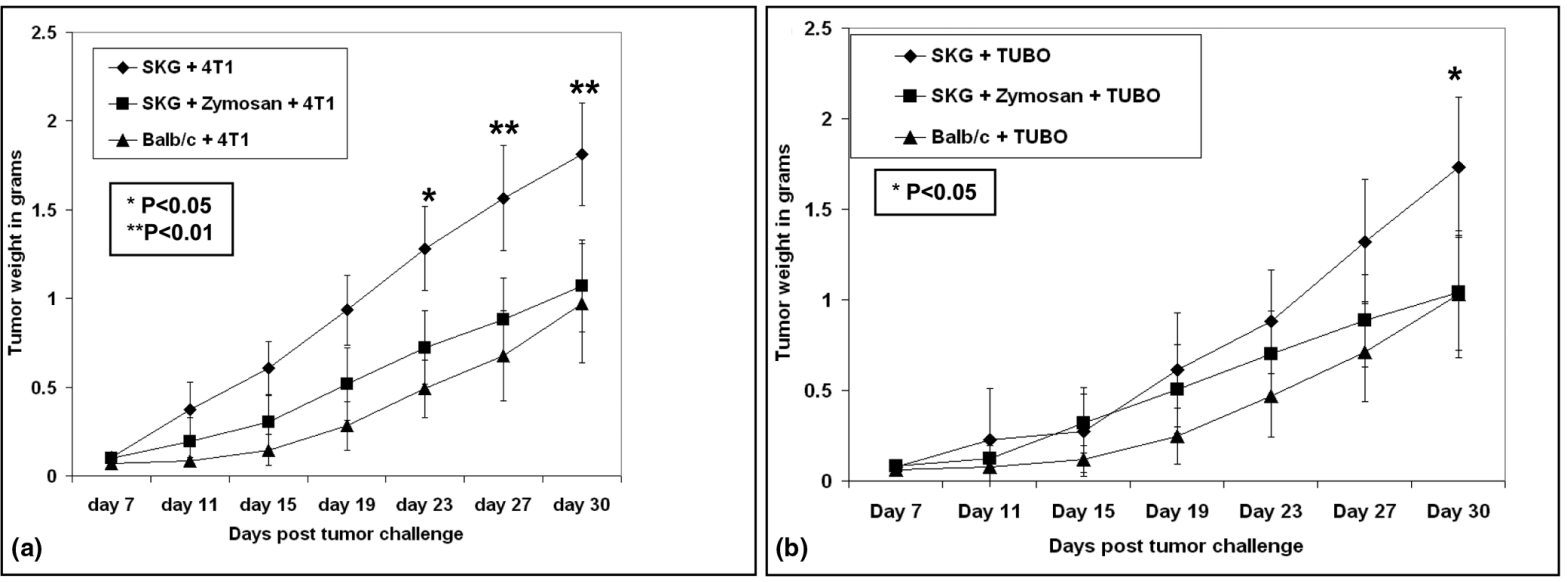
Kinetics of tumor growth in arthritic versus non-arthritic mice. **(a) **Mice injected with 4T1 cells; **(b) **mice injected with TUBO cells. Compared with the non-arthritic Balb/c mice, a significant increase in tumor burden was observed in SKG mice challenged with 4T1 (** *P *< 0.01) and TUBO (* *P *< 0.05) cells. Note: All tumor cells were injected in the mammary fat pad and sacrificed four-weeks post tumor challenge. n = 10 mice per group.

Next, we questioned if the primary tumor burden was affected by the arthritic milieu in the SKG mice. In SKG mice, both 4T1 and TUBO primary tumor burden was significantly higher compared with the non-arthritic Balb/c mice (Figures 2a and 2b ** *P *< 0.01, * *P *< 0.05). However, to our surprise the primary tumor burden in the SKG + zymosan mice was not significantly different from the Balb/c control mice (Figures 2a and 2b) suggesting that the primary tumor burden was not further influenced by zymosan-induced arthritis. We do not understand at this time why the tumor burden in SKG + zymozan and Balb/c were similar. Nevertheless, secondary metastasis to the lungs and bone was significantly altered in both the SKG and SKG + zymosan mice as discussed below.

**Figure 3:**
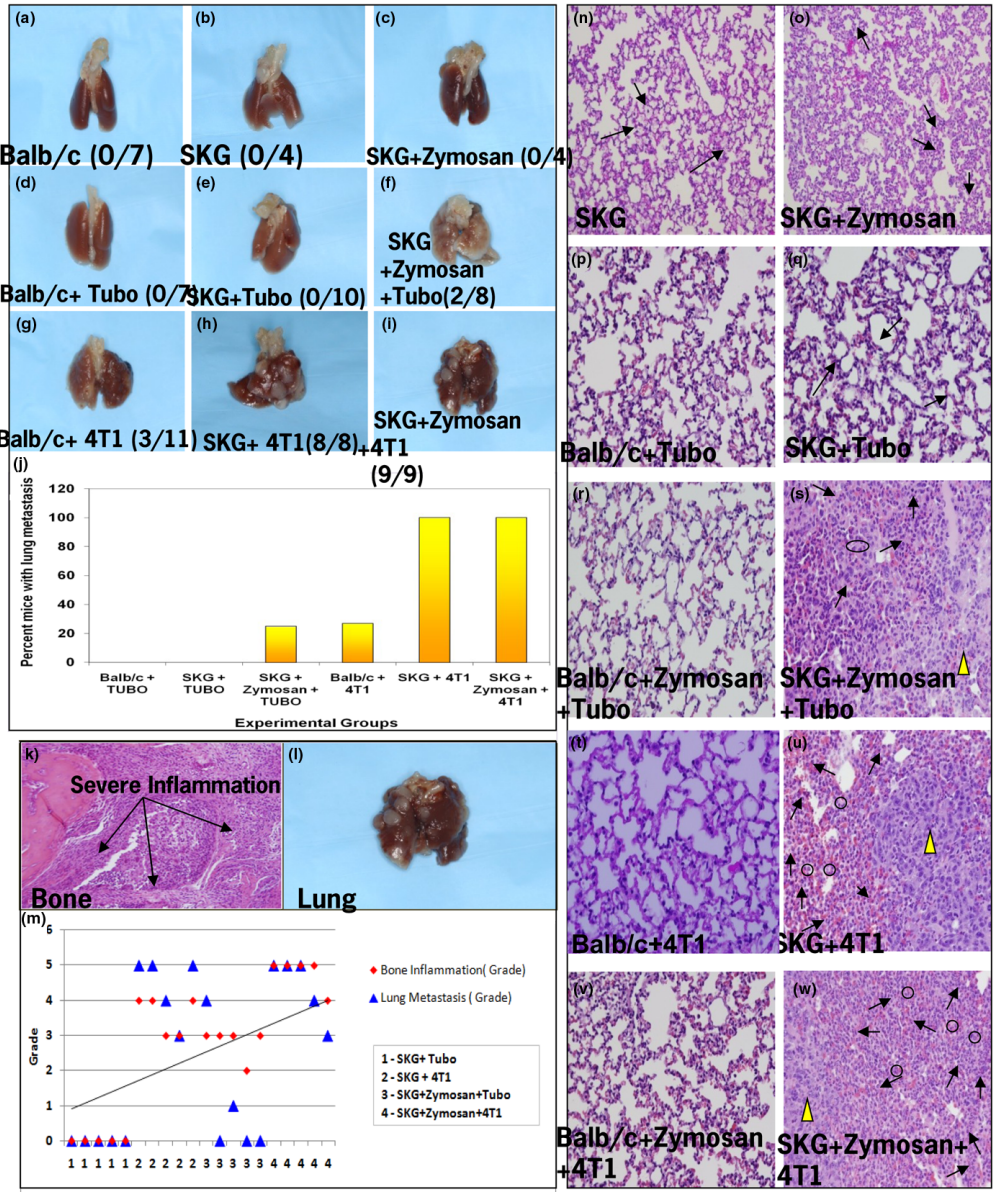
Autoimmune arthritis-related inflammation in the lungs is associated with a three-fold increase in the number of lung metastatic lesions in the SKG mice. Representative images of lungs from **(a) **Balb/c; **(b) **SKG; **(c) **SKG + Zymosan A; **(d) **Balb/c injected with TUBO cells; **(e) **SKG without Zymosan A + TUBO cells; **(f) **SKG + Zymosan A + TUBO cells (2/8 mice developed lung lesions); **(g) **Balb/c + 4T1 cells (3/11 mice developed lung lesions); **(h) **SKG without Zymosan A + 4T1 cells (8/8 mice developed severe lung metastasis); **(i) **SKG + Zymosan A + 4T1 cells (9/9 mice developed severe lung metastasis). **(j) **Percentage of mice that developed lung metastasis. **(k and l) **H&E of bone section showing high inflammation and image of lung with metastasis from the same 4T1-bearing SKG mouse. **(m) **A statistical analysis for correlation of bone inflammation and lung metastasis. The correlation coefficient for the 4T1 tumor-bearing SKG and SKG + Zymosan mice was determined to be 0.93 and 0.90. Correlation for TUBO-bearing mice was not calculated. **(n to w) **H&E staining of the lung sections from **(n) **SKG (moderate infiltration); **(o) **SKG + Zymosan A (moderate infiltration). **(p) **Balb/c + TUBO (no inflammation); **(q) **SKG + TUBO (moderate infiltration); **(r) **Balb/c + Zymosan A + TUBO; **(s) **SKG +Zymosan A + TUBO (moderate infiltration)**(t) **Balb/c + 4T1 (no infiltration); **(u) **SKG + 4T1 (severe infiltration); **(v) **Balb/c + Zymosan A + 4T1 (no infiltration); and **(w) **SKG + Zymosan A + 4T1 (severe infiltration). The solid filled arrow represents lung metastatic lesions, unfilled arrow represents neutrophils and unfilled circles represent macrophages. All images taken at 200 × magnification.

We observed a three-fold increase in the incidence of lung metastasis in the 4T1 tumor-bearing SKG and SKG + zymosan mice compared with the 4T1 tumor-bearing Balb/c mice (Figures 3a to 1j). All of the SKG (8/8 mice) and SKG + zymosan (9/9 mice) mice developed lung metastasis with the 4T1 tumors compared with only 27% (3/11 mice) of the Balb/c mice (Figure 3j). The number and size of the lung lesions were also found to be significantly higher in the SKG versus the Balb/c mice (data not shown). This result is especially significant because it represents true metastasis arising from the primary mammary gland tumor. Lungs from TUBO-challenged mice show no metastatic lesions in the Balb/c or SKG mice, albeit 2/8 SKG + zymosan mice developed lung lesions with TUBO cells (Figures 3d to 3f, and 3j). There were no metastatic lesions in lungs of control Balb/c, SKG, and SKG-zymosan mice without tumor challenge (Figures 3a to 3c, and 3j). A representative lung from each experimental group is shown (Figure 3a to 3i). It must be noted that the difference in metastasis was certainly not due to primary tumor size, because the zymosan group and Balb/c group had no difference in primary tumors but had significant difference in metastasis. In addition, the primary tumor weight of TUBO and 4T1 was similar but metastasis was significantly different. In any case, the smallest tumor was still pretty large, that is, 1 g or 1000 mgs.

To investigate a mechanism of the increased lung metastasis, we first examined the lung histology from the various mice. It became apparently clear from the H&E staining that all lungs that developed metastasis were packed with inflammatory cellular infiltrates characterized by prominent neutrophilic and granulocytic cells and activated macrophages (Figure 3n to 3w). Along with the cellular infiltrates, epithelial metastatic lesions were identified in the lungs as indicated by the solid yellow arrowheads (Figures 3s, u, and 3w). Infiltration in the SKG + 4T1 and SKG + zymosan + 4T1 lungs (Figures 3u and 3w) was significantly higher than in SKG (Figure 3n), SKG + zymosan (Figure 3o), and SKG + TUBO (Figure 3q) mice. Infiltration in the SKG + 4T1 or SKG + zymosan + 4T1 lungs was significantly higher than in 4T1 bearing Balb/c mice ± zymosan mice (compare Figures 3t and 3v with 3u and 3w). Interestingly, the TUBO and 4T1-bearing Balb/c showed completely normal lung architecture with no infiltration (Figures 3p and 3r) and in contrast the SKG + zymosan + TUBO showed significant infiltration (compare Figure 3r with 3s) but only in the mice that developed metastasis (Figure 3j). Data suggest a high degree of correlation (correlation coefficient of 0.99) between the level of cellular infiltration in the lungs and the severity of lung metastasis.

The next obvious question was whether the severity of lung metastasis follows the severity of arthritis. A representative lung and bone section from a 4T1 tumor-bearing SKG mouse is shown in Figures 3k and 3l, in which severe inflammation in the fore and hind limbs correlated with severe metastatic lung lesions. Significant correlation exists between severity of joint inflammation (arthritis) and the development of lung metastasis in the 4T1 tumor-bearing SKG and SKG + zymosan mice (correlation coefficient of 0.93 and 0.90 respectively; Figure 3m).

**Figure 4:**
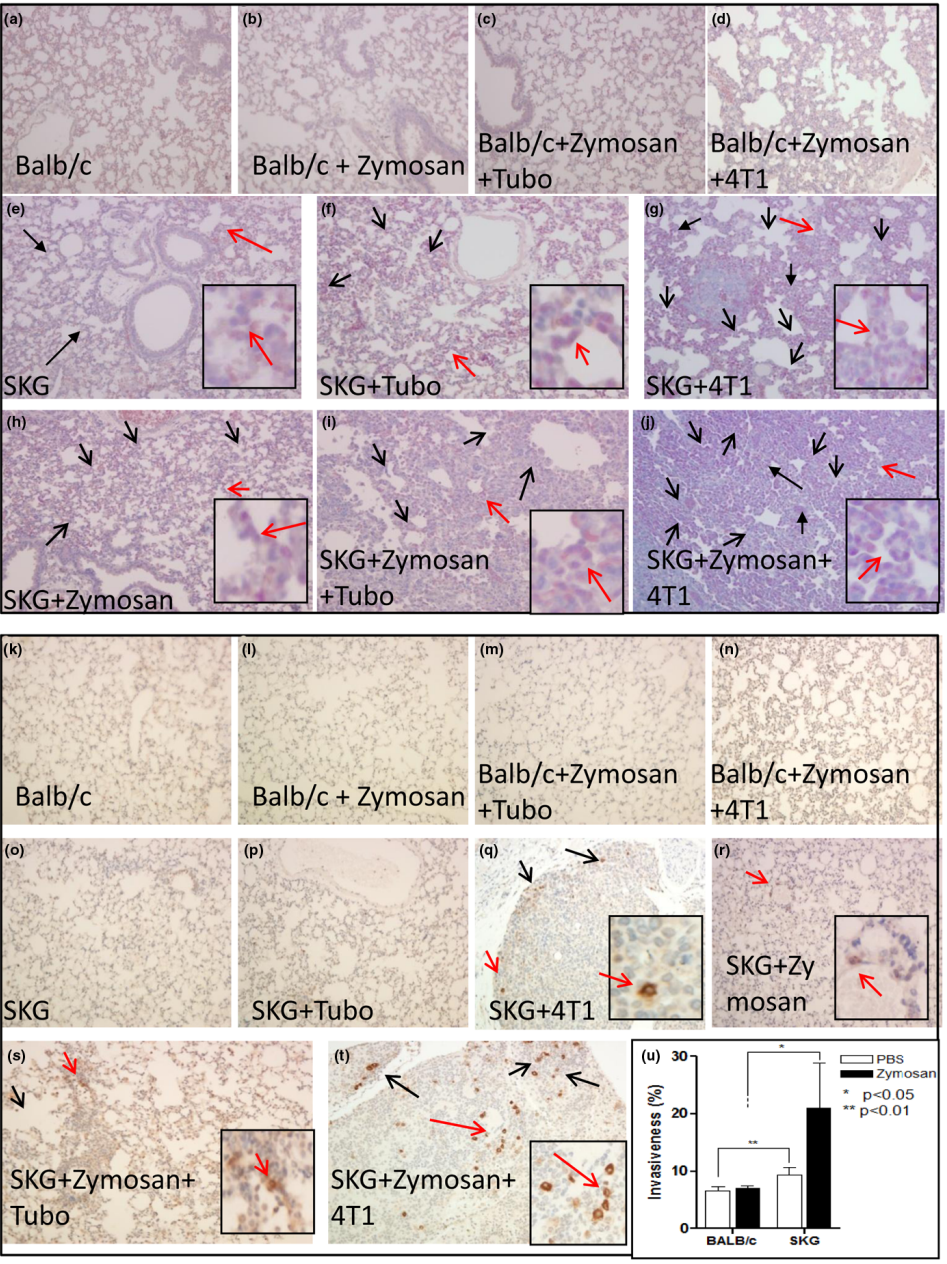
Increased infiltration of neutrophils and macrophages in the arthritic SKG mice versus the non-arthritic Balb/c mice. **(a to j) **Naphthol AS-D chloroacetate esterase staining of the lungs staining for neutrophils. **(a) **Balb/c; **(b) **Balb/c + Zymosan; **(c) **Balb/c + Zymosan + TUBO; **(d) **Balb/c + Zymosan + 4T1; **(e) **SKG (moderate infiltration of neutrophils); **(f) **SKG + TUBO(moderate infiltration); **(g) **SKG + 4T1(severe infiltration of neutrophils); **(h) **SKG + Zymosan(moderate infiltration); **(i) **SKG + Zymosan + TUBO (moderate infiltration); **(j) **SKG + Zymosan +4 T1(severe infiltration of neutrophils). No infiltration of neutrophils observed in any other experimental groups. **(k to t) **F4/80 staining of the lungs showing macrophages. **(k) **Balb/c; **(l) **Balb/c + Zymosan; **(m) **Balb/c + Zymosan + TUBO; **(n) **Balb/c + Zymosan + 4T1; **(o) **SKG; **(p) **SKG + TUBO; **(q) **SKG +4T1 (increased macrophages); **(r) **SKG + Zymosan (few macrophages); **(s) **SKG + Zymosan + TUBO (few macrophages); **(t) **SKG + Zymosan + 4T1 (increased infiltration of macrophages). No infiltrating macrophages observed in any other experimental groups. **(u) **BAL fluid from arthritic lungs caused significant increase in invasiveness of the 4T1 cells as compared with the BAL from non-arthritic lungs. All images taken at 200 × magnification. All slides were examined by the histology personnel and Dr Gruber at the Carolinas Medical Center confirming neutrophil infiltration.

To further demonstrate the chemotactic microenvironment in the lungs of arthritic versus non-arthritic mice, specific staining for neutrophils and macrophages was conducted. Data showed significantly increased levels of neutrophilic infiltration in the non-tumor-bearing SKG and SKG + zymosan lungs as compared with the Balb/c and Balb/c + zymosan lungs (Figures 4a, b, e, and 4h) suggesting a possible role for the infiltrating neutrophils in attracting the 4T1-tumor cells to the lungs of the arthritic mice. Not surprising, the neutrophilic infiltration was severely elevated with the 4T1 cells but not as much with the TUBO cells (compare Figures 4g and 4j with 4f and 4i) implying that the 4T1 metastasis further creates a stronger chemotactic microenvironment in the lungs of the arthritic mice to promote additional neutrophilic infiltration. In the Balb/c mice, neither zymosan injection nor tumor challenge caused any neutrophilic infiltration in the lungs (Figures 4c and 4d). Although neutrophilic infiltration was most prominent, we detected a few infiltrating macrophages in the SKG + zymosan group even without any tumor (Figure 4r), which was also considerably increased with 4T1 metastasis (Figures 4q and 4t).

### BAL fluid from arthritic mice is highly chemotactic for the 4T1 cells *in vitro*

Although the data above does not prove, it certainly suggests that the increased cellular infiltration in the lungs of the arthritic mice versus the non-arthritic mice may be one of the underlying mechanisms for the increased rate of metastasis observed in the arthritic mice (Figure 3j). To substantiate the chemotactic potential of the arthritic lung, BAL from lungs of the arthritic and non-arthritic mice was used as the chemotactic factor in an *in vitro *trans-well invasion assay with the 4T1 cells in the top chamber and BAL in the bottom chamber. Data clearly shows that BAL collected from the SKG + zymozan or SKG lungs significantly augment the invasiveness of the 4T1 cells as compared with the BAL from Balb/c or Balb/c + zymosan lungs (Figure 4u). Preliminary analysis of the BAL from SKG + zymozan, and SKG mice showed higher levels of pro-matrix metalloproteinase (MMP)-9, macrophage colony stimulating factor (M-CSF) and IL-17 compared with the BAL from Balb/c + zymozan mice (data not shown).

### Increased expression of pancytokeratin positive epithelial cells in the arthritic bones coupled with increased expression of COX-2

**Figure 5:**
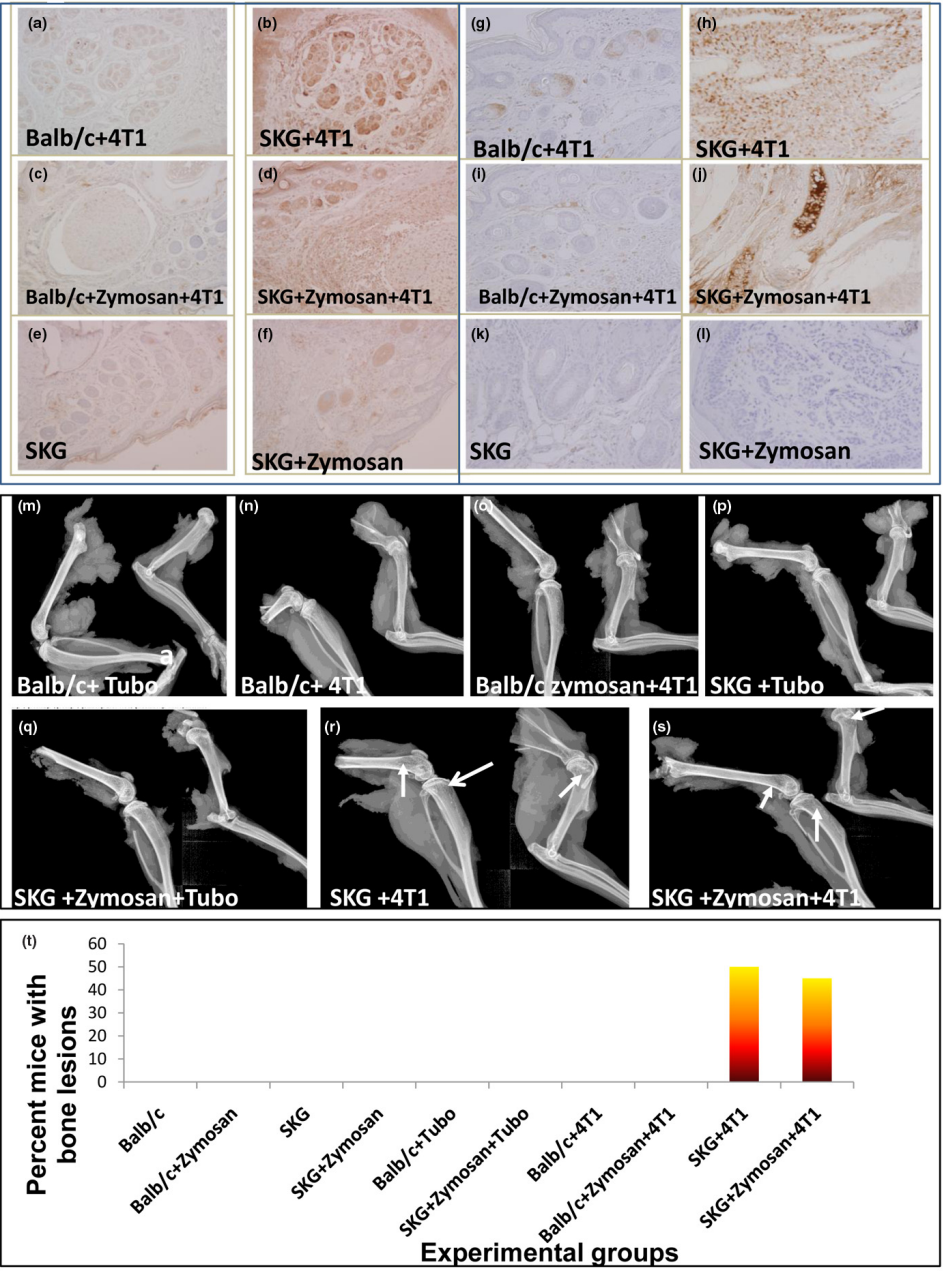
Increased expression of COX-2 and pancytokeratin in the bones of arthritic mice challenged with 4T1 cells. Bone sections from various experimental groups (indicated in the Figure) stained for COX-2 **(a to f) **and pancytokeratin **(g to l)**. Brown staining represents positive staining. All images taken at 200 × magnification. **(m to s) **Representative Faxitron x-ray images of bones. **(m) **Balb/c + TUBO; **(n) **Balb/c + 4T1; **(o) **Balb/c + Zymosan + 4T1; **(p) **SKG + TUBO; **(q) **SKG + Zymosan + Tubo; **(r) **SKG + 4T1 (showing small radiolucencies in distal femur, proximal tibia, and head of humerus indicating apparent osteolytic bone lesions); **(s) **SKG + Zymosan A + 4T1 (showing radiolucencies in the distal femur, proximal tibia and head of humerus possibly due to osteolytic bone lesion). Six to seven mice from each group were examined using the faxitron x-ray. No lytic or sclerotic lesions were observed in any other experimental group. **(t) **Percentage of mice that developed bone metastasis.

We speculate that the cellular infiltration in the lungs may have greatly facilitated the recruitment of breast cancer cells to the site (Figures 3 and 4), so we examined if high cellular infiltration in the bones of the arthritic mice had a similar effect. We have already established increased cellular infiltration in the bones of arthritic versus non-arthritic mice (Figures 1h to 1n), which was also characterized by neutrophilic infiltration (data not shown) and osteoclast formation (Figures 1o to 1t). We now examined the levels of pro-inflammatory factor, COX-2 in the bone that may create a milieu conducive for increased bone metastasis. Inflammation in the joints is usually represented by high levels of COX-2, which also play a critical role in fostering tumor growth and metastasis [15-18]. In comparison to the non-arthritic Balb/c mice with tumor challenge, the expression of COX-2 in the bones of the arthritic mice was significantly greater even without any tumor (Figures 5a, c, e, and 5f), which was undeniably augmented when these arthritic mice were challenged with the 4T1 tumors (Figures 5b and 5d) but not with TUBO (data not shown). To directly test the presence of epithelial (tumor) cells in the inflamed bones, sections of the bone were stained with pancytokeratin antibody. Increased expression of pancytokeratin-positive areas was only evident in the bones of the 4T1 tumor-bearing arthritic mice (Figures 5h and 5j). Bones from all other experimental groups were negative (Figures 5g, i, k, and 5l). Brown staining represents COX-2 and pancytokeratin positivity. Bones from eight mice were examined with similar staining patterns. Thus, we hypothesize that the pro-inflammatory microenvironment in the arthritic bone (Figures 1 and 5) may boost the recruitment of the 4T1 cells and that the 4T1 cells in turn augments the severity of arthritis (Figure 1); thus creating a highly conducive microenvironment for the 4T1 tumors to further proliferate.

### Increased 4T1 bone lesions in the arthritic versus non-arthritic mice

Bones from six to seven mice were analyzed by x-ray imaging for osteolytic lesions. Representative images are shown in Figures 5m to 5s. Osteolytic and/or sclerotic bone lesions were determined in 50% (3/6) of SKG mice and 43% (3/7) of the SKG + zymosan mice challenged with the 4T1 metastatic tumor cells (Figures 5r, s, and 5t). These bones show distinct radiolucencies in the distal femoral diametaphysis, proximal tibia and head of humerus indicating apparent osteolytic bone lesion (as indicated by the arrows; Figures 5r and 5s). In contrast, none of the Balb/c mice challenged with 4T1 or TUBO tumors developed bone lesions (Figures 5m to 5o) nor did any of the SKG mice challenged with TUBO cells (Figures 5p and 5q). The results confirm that bone lesions only occur in the pro-arthritic and arthritic bones but not in non-arthritic bones (Figure 4t). Taken together, data from Figures 1 and 5 imply that cellular infiltration and osteoclast formation along with increased COX-2 may provide a conducive microenvironment for 4T1 tumor cells to metastasize to the bone, which further augments cellular infiltration and osteoclast formation associated with increased bone damage completing the vicious cycle of osteolytic bone metastasis [19].

### M-CSF, IL-6, IL-17, TNF-α, and VegF may be the underlying factors responsible for the increased metastasis in the arthritic mice

**Figure 6:**
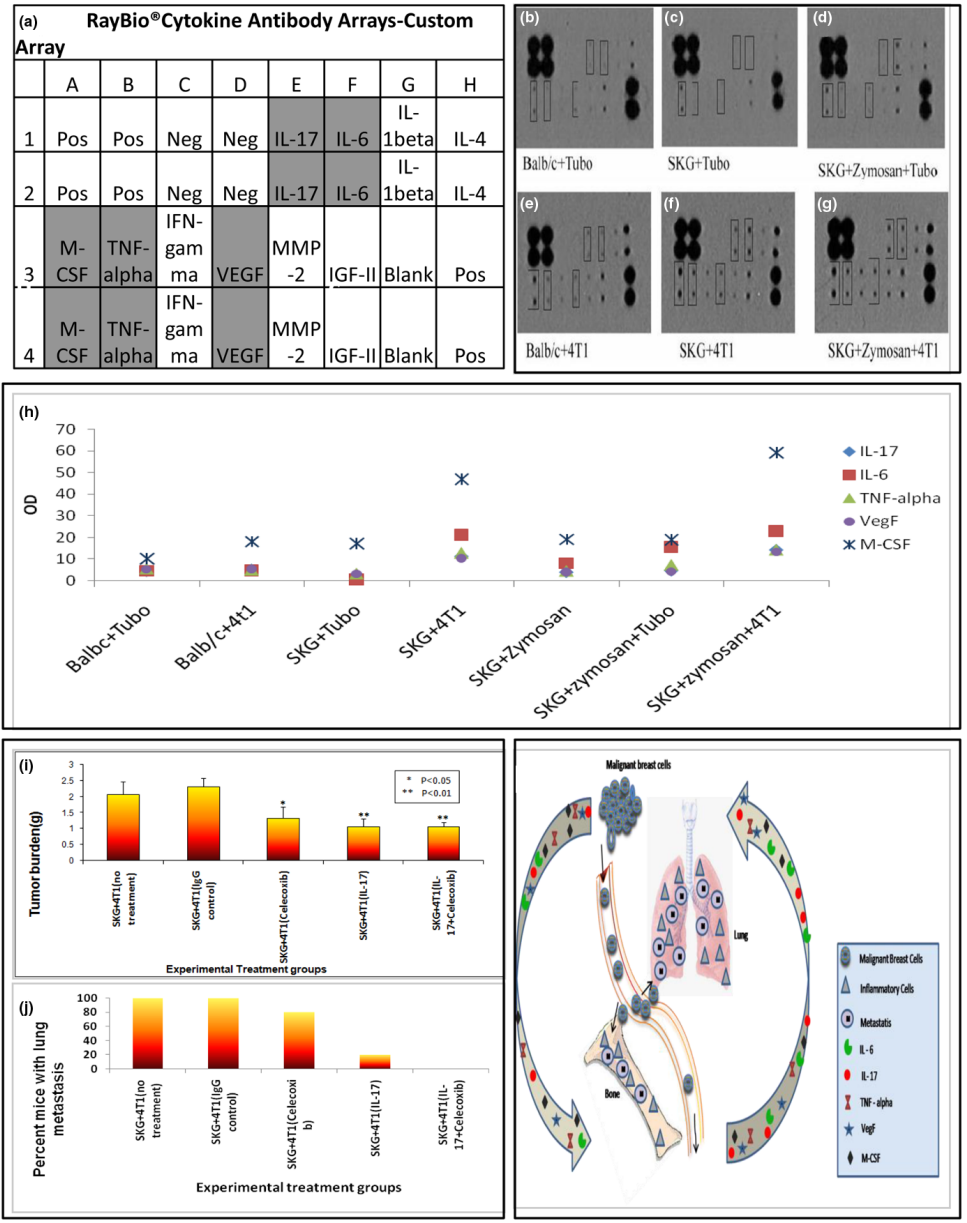
Serum analysis of cytokines revealed up regulation of several cytokines in the arthritic mice compared with the non-arthritic mice, and treatment with IL-17 antibody and celecoxib completely prevented formation of lung metastasis in the SKG mice. **(a) **Array template of the cytokines on the membrane. Up-regulation of macrophage colony stimulating factor (M-CSF), TNF-alpha, IL-17, vascular endothelial growth factor (VegF), and IL-6 is observed and highlighted in gray on the template **(b to g) **Membrane dot blot representing circulating cytokine levels in various groups of mice (indicated in the Figure). Boxes are drawn around the cytokines that were up-regulated. **(h) **A graphical representation of the up-regulated cytokines observed on the membrane blots based on densitometry analysis (n = 4 mice). **(i) **Significant reduction in tumor burden in mice treated with celecoxib (*P *< 0.05), α-IL-17 antibody (*P *< 0.01) or α-IL-17 antibody + celecoxib (*P *< 0.01) as compared with the untreated or IgG isotype group; **(j) **Percentage of mice that developed lung metastasis. **(k) **Schematic model depicting the vicious interaction between metastatic breast tumors and the inflammatory microenvironment in the bone and lung due to autoimmune arthritis (AA). During arthritis, inflammatory cell infiltrate in the bones and lungs trigger the release of pro-inflammatory cytokines. These cytokines act directly or indirectly on the primary breast tumor cells enhancing their metastatic ability. The ensuing damage to the bones and the large amounts of inflammatory cells in the bones and lungs of the arthritic mice allows retention and growth of the tumor cells in the site. The final distribution of metastasis reflects the relative abundance of inflammation in the organs. Thus, we hypothesize that the increase in some of the proinflammatory cytokines such as IL-17 may be the underlying factor responsible for the increased metastasis.

To determine the possible mechanism that drives the 4T1 cells to become more metastatic in the arthritic model, we evaluated the circulating levels of pro-inflammatory cytokines and chemokines in the sera of the arthritic versus the non-arthritic mice. A custom mouse cytokine array was designed to test the sera for the presence of 10 cytokines known to be associated with osteolysis as well as tumor growth and metastasis [19]. These included M-CSF, TNF-α, interferon-gamma (IFN-γ), vascular endothelial growth factor (VegF), IL-17, MMP-2, IL-6, Insulin-like growth factor-II (IGF-II), IL-1β, and IL-4 (Figure 6a). Compared with the 4T1 tumor-bearing Balb/c mice, significant increase in the levels of M-CSF (1.5 fold), IL-17 (1.5 fold), TNF-α, VegF, and IL-6 (1.2 fold) was observed in the 4T1 tumor-bearing arthritic mice (Figures 6e to 6g). A graphical representation of the values from the densitometric analysis is provided in Figure 6h. In the TUBO-bearing mice, the increase was non-significant between Balb/c and the arthritic mice (Figures 6b to 6c). Serum from SKG + zymosan mice (without tumor challenge) was used as control and the cytokine levels was found to be similar to that found in sera of SKG + zymosan + TUBO and SKG + TUBO mice (Figure 6h) implying that the increase in the cytokine levels was not just a function of zymosan injection but was driven by a combination of arthritic milieu and metastatic 4T1 tumors but not by either condition alone.

### Treatment with anti-IL17 treatment in combination with celecoxib prevents 4T1-associated metastasis in the SKG mice

IL-17 was elevated in our model, and because it is an emerging therapeutic target for cancer metastasis and arthritis [20-26], we tested if neutralizing IL-17 will be an effective therapy against the development of secondary metastasis in our arthritic model. We elected to conduct treatment with an anti-IL-17 antibody either alone or in combination with celecoxib, a selective COX-2 inhibitor, in the SKG mice with established 4T1 tumors. The rationale for using a specific COX-2 inhibitor was that these drugs including celecoxib were originally developed for treating arthritis but have now been shown to have significant anti-cancer properties [27,28]. Established 4T1 tumor burden was significantly reduced in mice treated with celecoxib (*P *< 0.05), α-IL-17 antibody (*P *< 0.01), or celecoxib + α-IL-17 antibody (*P *< 0.01) when compared with untreated or control antibody + vehicle (DMSO) treated mice (Figure 6i). More importantly, mice treated with the combination of celecoxib + α-IL-17 antibody were completely spared of lung metastasis (Figure 6j.) and only 10% of the mice (1/10 mice) in the α-IL-17 antibody group developed lung metastasis compared with 80% (8/10 mice) in the celecoxib-treated and 100% (10/10 mice) in the untreated or control antibody + vehicle-treated mice (Figure 6j).

## Discussion

A significant increase in breast cancer-associated secondary metastasis to the lungs and bones were observed in the arthritic versus the non-arthritic mice (Figures 3 and 5) along with increase in primary tumor burden (Figure 2). In addition, the metastatic breast cancer 4T1 cells accentuate the severity of bone destruction and lung inflammation in the arthritic mice creating a favorable microenvironment for the tumor cells to grow (Figures 1, 3, and 4). We therefore suggest that chronic inflammation in the bone and lung caused by AA and the subsequent increase in circulating levels of proinflammatory cytokines may be the underlying mechanism for the increased metastasis (Figure 6). This is further substantiated when treatment with celecoxib + αIL-17 antibody completely protected the arthritic mice from 4T1 metastasis (Figure 6j), as well as significantly reduced established primary tumor (Figure 6i).

Compared with the non-arthritic Balb/c mice, the lungs of the arthritic mice expresses high levels of cellular infiltrates mostly characterized by neutrophils and some macrophages even before any tumor challenge (Figures 4h and 4r) suggesting a pro-inflammatory milieu that may be responsible for attracting the 4T1 metastatic cells to the lungs as demonstrated in the *in vitro *invasion assay (Figure 4u). Similar low-level inflammation (as characterized by COX-2 staining and cellular infiltration) is also observed in the bones of the arthritic mice prior to tumor challenge, which is completely absent in the non-arthritic Balb/c bones (Figures 1 and 5). Once the 4T1 cells home to the lungs or bones, the level of interstitial inflammatory cellular infiltrates is exponentially increased characterized by prominent neutrophils and macrophages (Figures 1, 3, and 4). These pro-inflammatory leukocytes alone can facilitate tumor cell extravasation and promote metastasis [29]. Thus, it is not surprising that we saw an increase in pro-inflammatory and pro-angiogenic cytokines in the serum of these 4T1 tumor-bearing arthritic mice versus the Balb/c non-arthritic mice (Figure 6h).

This is the first study that undoubtedly establishes a correlation between the pro-inflammatory cell recruitment in the lungs during AA and the homing of the circulating tumor cells in the inflamed lungs (Figures 3 and 4). In the bone, once the metastatic breast cancer cells get attracted to the inflamed bone milieu (Figures 1 and 5), they produce unknown factors that directly or indirectly induce osteoclast formation (Figure 1) causing bone resorption and release of growth factors from the bone matrix which in turn stimulates further tumor growth (schematically illustrated in Figure 6k) and reviewed in [19]. This reciprocal interaction between breast-cancer cells and the bone microenvironment results in a vicious cycle that increases both bone destruction and increases tumor burden and metastasis (Figure 6k) [19].

As in human AA, cytokines play an essential role in the development of arthritis in the SKG mice [9,30]. Several cytokines have been implicated in the mechanism of synovial cell activation and joint destruction in AA [31]. In our study, serum analysis of cytokine proteins revealed higher expression of M-CSF, IL-17, IL-6, TNF-α, and VegF in the arthritic mice only when challenged with 4T1 metastatic breast cancer cells (Figure 6h). Elevated serum M-CSF predicts reduced survival in metastatic breast cancer patients [32,33]. The M-CSF produced by breast cancer cells and surrounding stroma increases osteoclast formation and maturation and enhances the expression of stromal RANK ligand, both of which increase osteolytic bone degradation [34]. IL-17 has been identified as a crucial cytokine for osteoclastic bone resorption in AA patients [24]. IL-17 acts on osteoblasts by stimulating COX-2-dependent prostaglandin E_2 _(PGE_2_), and osteoclast differentiation factor, which differentiates osteoclast progenitors into mature osteoclasts, causing bone resorption. PGE_2 _interacts with its eicosanoid receptors to induce the damage [24]. IL-17 is also known to be upregulated in breast and other cancers [35-37]. IL-6 is an autocrine and paracrine growth factor for several cancers, including breast cancer [38,39] and stimulates cancer cell growth and contributes to recurrence and metastasis in breast cancer [40]. TNF-α is a known pro-inflammatory cytokine and generates an autocrine tumor promoting network in ovarian and breast cancer [41]. VegF is known to play a critical role in vasculogenesis, angiogenesis, and metastasis [42-44]. Upregulation of these cytokines may therefore account for the enhanced breast cancer-associated secondary metastasis as well as accentuating the severity of arthritis in the SKG mice. Thus, blocking these pro-inflammatory cytokines may hold promise in reducing the severity of both diseases. Indeed when we blocked the IL-17 pathway, we observed a significant reduction in established primary tumor burden and even more significant reduction in rate of metastasis (Figures 6i and 6j). The effect was even greater when IL-17 blockade was combined with blocking the COX-2 pathway where none of the mice treated with the combination developed any metastasis (Figure 6j).

Inflammation is a critical hallmark of arthritis and tumor progression [2,45-47]. Many processes that occur during arthritis also occur during tumorigenesis. There is increased vascularity in both, and there are common cytokines and growth factors that are regulated in both. The microenvironment in the tumor and arthritis is largely orchestrated by inflammatory cells and growth factors [48,49]. Thus, it is not unlikely that the two diseases commonly co-exist in women. Our study begins to evaluate whether these two disease states molecularly interact and feed off each other. Data from these studies were further substantiated in our preliminary study using a spontaneous metastatic breast cancer model (mice expressing the polyoma middle T antigen driven by the MMTV promoter) [50] that develop collagen-induced arthritis [51]. Similar increases in bone and lung metastasis is noted in this model as well (unpublished data). These studies may have important clinical implications, especially in the prevention of secondary metastasis, in designing combination drug regimens, and as a diagnostic risk-assessment tool.

## Conclusions

Our data provide clear evidence for the first time that breast cancer-associated secondary metastasis is significantly increased in pro-arthritic and arthritic conditions and that blocking the IL-17 and COX-2 pathway may significantly reduce the rate of metastasis.

## Abbreviations

AA: autoimmune arthritis; BAL: bronchoalveolar lavage; COX: cyclo-oxygenase; FBS: fetal bovine serum; H&E: hematoxylin and eosin; IFN: interferon; Ig: immunoglobulin; IGF: insulin-like growth factor; IL: interleukin; ip: intraperitoneal; M-CSF: macrophage colony stimulating factor; MMP: matrixmetalloproteinase; NaCl: sodium chloride; PBS: phosphate buffered saline; PGE_2_: prostaglandin E_2_; SF: serum free; TNF: tumor necrosis factor; VegF: vascular endothelial growth factor.

## Competing interests

The authors declare that they have no competing interests.

## Authors' contributions

LDR designed and carried out the experiments, and wrote the manuscript. LP and TT helped with the dissections and endpoints. JS helped with the collection of BAL and designing the *in vitro *invasion assay. HEG conducted and interpreted some of the bone-associated histology and x-ray imaging. PM is the principal investigator of the laboratory in which the research was performed and contributed to the interpretation of the data and writing of the manuscript.

## Authors' information

Dr Pinku Mukherjee, PhD, Irwin Belk Distinguished Professor of Cancer Research, Department of Biology, University of North Carolina, Charlotte, NC. Dr Mukherjee is a trained tumor immunologist and has been an Associate Professor at the Department of Immunology in Mayo School of Medicine prior to moving to UNCC in September 2008. Dr Mukherjee has worked on Breast Cancer for the past 20 years.

Dr Lopamudra Das Roy, PhD, Postdoctoral research associate, Department of Biology, University of North Carolina, Charlotte, NC. Dr Roy has received funding for her work in Breast Cancer Research from The US Department of Defense Breast Cancer Foundation

Latha Pathangey, MSc, senior technologist, Mayo Clinic Arizona, Department of Biochemistry/Molecular Biology, Scottsdale, AZ. Ms Pathangey has worked with Dr Mukherjee for the past five years at Mayo Clinic.

Teresa Tinder, BSc, senior technologist, Department of Biology, University of North Carolina, Charlotte, NC. Ms Tinder has worked with Dr Mukherjee for the past 10 years.

Jorge L. Schettini, PhD, Research Assistant Professor, Department of Biology, University of North Carolina, Charlotte, NC. Dr Schettini is a trained immunologist and has been working with Dr Mukherjee for the past three years.

Helen Gruber, PhD, Director, Biology Division, Department of Orthopedic Surgery, Carolinas Medical Center, Charlotte, NC. Dr Gruber has over 25 years of experience in the area of bone pathology and osteoarthritis and bone metastasis.
